# Supplementary material for: Connecting virtual reality and ecology: a new tool to run seamless immersive experiments in R
Source: PeerJ Comput Sci. 2021 Jun 1;7:e544. doi: 10.7717/peerj-cs.544 (PMC8176535; doi:10.7717/peerj-cs.544)
Supplement: Supplemental Information 1 — Additional information about the r2vr package, associated codes and how people tested it. [file peerj-cs-07-544-s001.pdf]

Supporting Information
`r2vr`: Connecting virtual reality and ecology in `R`

Julie Vercelloni, Jon Peppinck, Edgar Santos-Fernandez, Miles McBain, Grace Heron, Tanya Dodgen, Erin E. Peterson, Kerrie Mengersen

Contents

R scripts for case studies - `r2vr` vignette 1

Screenshots of the online database 7

Training Document for participants 8
- Koala . . . 8
- Jaguar . . . 8
- Coral reef . . . 8

`r2vr` functions 9
- `start()` . . . 9
- `end()` . . . 9
- `pop()` . . . 9
- `go()` . . . 11
- `read()` . . . 13

R scripts for case studies - `r2vr` vignette

Interactions for Elicitation

Grace Heron, Jon Peppinck

2020-02-28

R objects for building A-Frame Scenes with Interactive Entities

New additions to `r2vr` now include interactive entities that store the outputs into a database. So far there are two question types. The first is a binary question with options for `1` or `0` as responses. The second is a multivariable response question with four answer options.

- `binary_question_scene()`
- `multivariable_question_scene()`

Case Study 1: Koalas

First you will need to install `ACEMS/r2vr` into your R library. Also you will need to set up a data base on db4freewith the same class name. For this example our class name is `koala`.

Next you will need to know your local IP address on your computer. You can find this by opening a command window (cmd) and running the command `ipconfig` and the IP address next to `IPv4 Address` is what you need. OR use `find_IP()` function in our `r2vr` package.

```
library(r2vr)
#>
#> Attaching package: 'r2vr'
#> The following objects are masked from 'package:stats':
#>
#>     end, start

## Other packages for data handling and visualisation
library(ggplot2)
library(reshape2)
library(knitr)
library(lubridate)
library(dplyr)

## Find the IPv4 Address
IPv4_ADDRESS <- find_IP()
```

Create experiment

You will need a folder of VR compatible images to use. Set up a variable that lists the paths to each image.

```
## Full path to image web locations
r2vr_pkg <- "https://cdn.jsdelivr.net/gh/milesmcbain/r2vr@master/inst"

## Append to get full paths to images
img_paths <- paste(r2vr_pkg,
                   c("/ext/images/koalas/Site_1.jpg",
                     "/ext/images/koalas/Site_2.jpg",
                     "/ext/images/koalas/Site_3.jpg",
```

```
                      "/ext/images/koalas/Site_4.jpg"),
                    sep = "")
```

For this experiment we wish to ask a question with a yes or no answer so we will use `binary_question_scene()`.

```
animals <- binary_question_scene(
  the_question = "Do you see any koalas in this image?", # Our question
  answer_1 = "Yes",  # Text for '1'
  answer_2 = "No",   # Text for '0'
  img_paths = img_paths, # Image paths
  IPv4_ADDRESS = IPv4_ADDRESS, # Our local IP address
  animal_class = "koala" # Class name for database
)
```

Let's start the VR server. Copy and paste your IP address into an internet browswer after running the below command.

```
start(IPv4_ADDRESS)
#> Fire started at 131.181.64.95:8080
```

Now the participant should be viewing the VR server in an internet browser on desktop or in a VR headset. Once the participant is immersed into the scene, we can now 'pop' the question.

```
pop()
```

The question and answer boxes should pop up on the displayed image. This command will only work once the scene is displayed. For the binary question, once an answer is selected it will be locked in so that answers are not duplicated for each participant for the same image.

Next we will want to move to the next image with the below command.

```
go(image_paths = img_paths, index = 2) # going to the second image
#> [1] "img2"
```

Again we pop the question and wait for the participant to answer.

```
pop()
```

We can also 'unpop' to remove the question and answer boxes.

```
pop(F)
```

When you are done with your image set, simply use the below command to close the server.

```
end()
#> [[1]]
#> NULL
```

Time for the exciting part which is to import our elicitation data. Note that the end of the url is our animal class (koala).

```
## Get data from database with API GET request
koala.df <- read(url = "https://test-api-koala.herokuapp.com/koala")
if(!is.null(nrow(koala.df))){
  koala.df$recordedOn <- lubridate::ymd_hms(koala.df$recordedOn, tz =
"Australia/Queensland")
  knitr::kable(tail(koala.df))
  ## Tidy dataframe for plotting
  koala.df <- dplyr::mutate(koala.df,
                            binary_response = dplyr::case_when(binary_response == 0 ~ "No",
                                                               binary_response == 1 ~
"Yes",
                                                               TRUE ~ "unknown"))

  ## Basic visualisation of data
  ggplot2::ggplot(koala.df, aes(fill = as.factor(binary_response), x = image_id)) +
    geom_bar(colour = "black") +
    theme_bw() +
```

```
    labs(fill = "Do you see any\nkoalas in this image?") +
    ggtitle("Koala presence response")
}
#> Date in ISO8601 format; converting timezone from UTC to "Australia/Queensland".
```

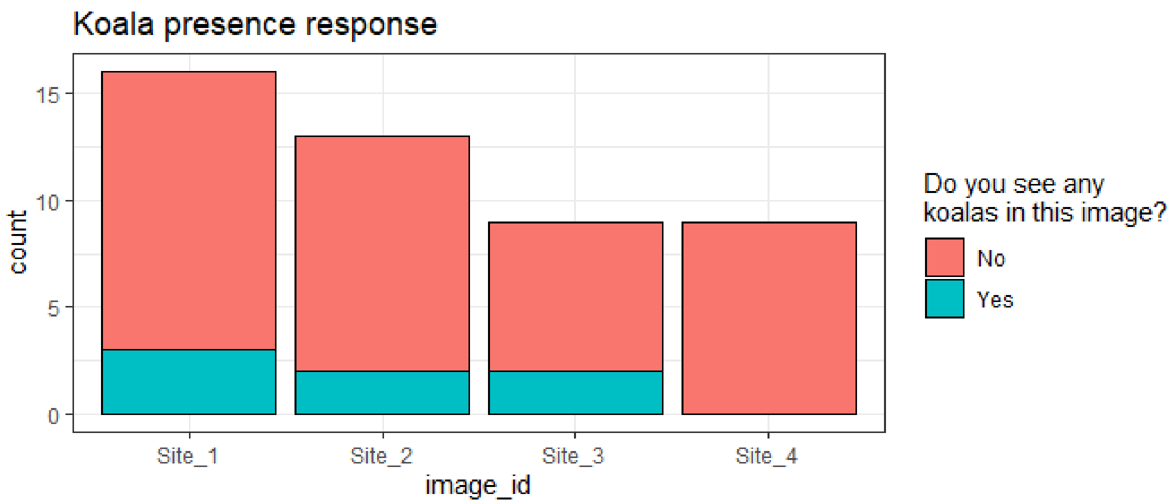

Case Study 2: The Great Barrier Reef

Repeat for reef case study.

```
## Define image paths
img_paths <- paste(r2vr_pkg,
                   c("/ext/images/reef/100030039.jpg",
                     "/ext/images/reef/120261897.jpg",
                     "/ext/images/reef/130030287.jpg",
                     "/ext/images/reef/130050093.jpg"),
                   sep = "")

## Create binary qestion scene for animals
animals <- binary_question_scene(
  the_question = "Do the live corals on this reef form a structurally complex habitat?",
  answer_1 = "Yes",
  answer_2 = "No",
  img_paths = img_paths,
  IPv4_ADDRESS = IPv4_ADDRESS,
  animal_class = "reef")

## Launch VR server
start(IPv4_ADDRESS)
#> Fire started at 131.181.64.95:8080

## Pop a question for first scene
pop()

## Move to new scene
go(image_paths = img_paths, index = 3)
#> [1] "img3"

## Don't forget to pop the question!
pop()

## Finish
end()
#> [[1]]
#> NULL
```

```r
## Get data from database with API GET request
reef.df <- read(url = "https://test-api-koala.herokuapp.com/reef")
## Check for empty data frame
if(!is.null(nrow(reef.df))){
  reef.df$recordedOn <-  lubridate::ymd_hms(reef.df$recordedOn, tz =
"Australia/Queensland")
  knitr::kable(tail(reef.df))
  ## Tidy dataframe for plotting
  reef.df <- dplyr::mutate(reef.df,
                           binary_response = dplyr::case_when(binary_response == 0 ~ "No",
                                                              binary_response == 1 ~ "Yes",
                                                              TRUE ~ "unknown"))

  ## Basic visualisation of data
  ggplot2::ggplot(reef.df, aes(fill = as.factor(binary_response), x = image_id)) +
    geom_bar(colour = "black") +
    theme_bw() +
    labs(fill = "Do the live corals\non this reef form a\nstructurally complex\nhabitat?")
+
    ggtitle("Structurally Complex Coral Habitat")
}
#> Date in ISO8601 format; converting timezone from UTC to "Australia/Queensland".
```

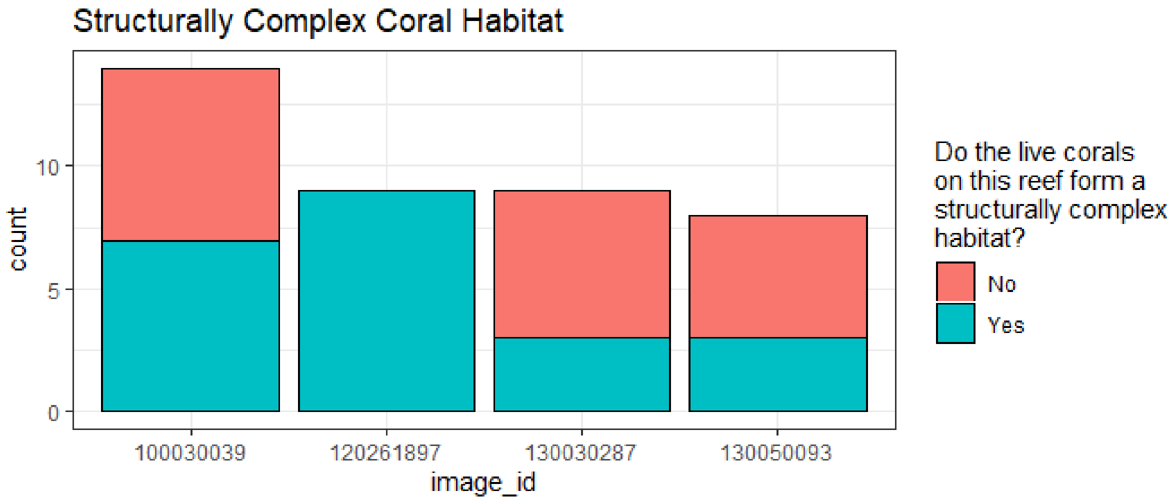

Case Study 3: Jaguars

```r
## Define image paths
img_paths <- paste(r2vr_pkg,
                   c("/ext/images/jaguars/WP14_360_002.jpg",
                     "/ext/images/jaguars/WP55_360_001.jpg",
                     "/ext/images/jaguars/WP56_360_001.jpg",
                     "/ext/images/jaguars/WP60_360_001.jpg"),
                   sep = "")

animals <- multivariable_question_scene(
  the_question = "Do you see any of these habitat features in this image? If you do see a
feature, click on the box to select it.",
  answer_1 = "Water",
  answer_2 = "Jaguar tracks",
  answer_3 = "Scratch marks",
  answer_4 = "Dense Vegetation",
  img_paths = img_paths, IPv4_ADDRESS)

## Launch VR server
start(IPv4_ADDRESS)
#> Fire started at 131.181.64.95:8080
```

```r
## Pop a question for first scene
pop(question_type = "multivariable")

## Move to new scene
go(image_paths = img_paths, index = 4, question_type = "multivariable")
#> [1] "img4"

## Don't forget to pop the question!
pop(question_type = "multivariable")

## Finish
end()
#> [[1]]
#> NULL

## Get data from database with API GET request
jaguar.df <- read(url = "https://test-api-koala.herokuapp.com/jaguar")
## Check for empty data frame
if(!is.null(nrow(jaguar.df))){
  jaguar.df$recordedOn <-  lubridate::ymd_hms(jaguar.df$recordedOn,
                                              tz = "Australia/Queensland")
  knitr::kable(tail(jaguar.df))
  ## Tidy dataframe for plotting
  jaguar.df <- dplyr::mutate(
    dplyr::filter(
      reshape2::melt(
        jaguar.df, measure.vars = c("option_1", "option_2", "option_3","option_4")),
      value == 1),
    response = dplyr::case_when(variable == "option_1" ~ "Water",
                                variable == "option_2" ~ "Jaguar tracks",
                                variable == "option_3" ~ "Scratch marks",
                                variable == "option_4" ~ "Dense vegetation",
                                TRUE ~ "unknown"))

  ## Basic visualisation of data
  ggplot2::ggplot(jaguar.df,aes(x = image_id, fill = response)) +
    geom_bar(position = "dodge", colour = "black") +
    facet_grid(cols = vars(image_id), scales = "free") +
    theme_bw() +
    labs(fill = "Observed\nhabitat\nfeatures") +
    ggtitle("Jaguar Habitat Features")
}
#> Date in ISO8601 format; converting timezone from UTC to "Australia/Queensland".
```

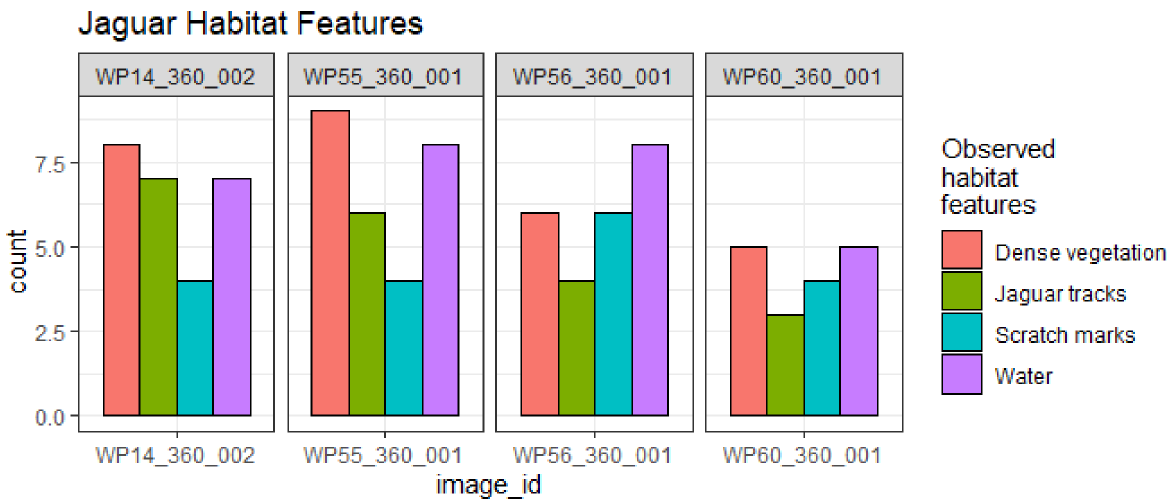

Screenshots of the online database

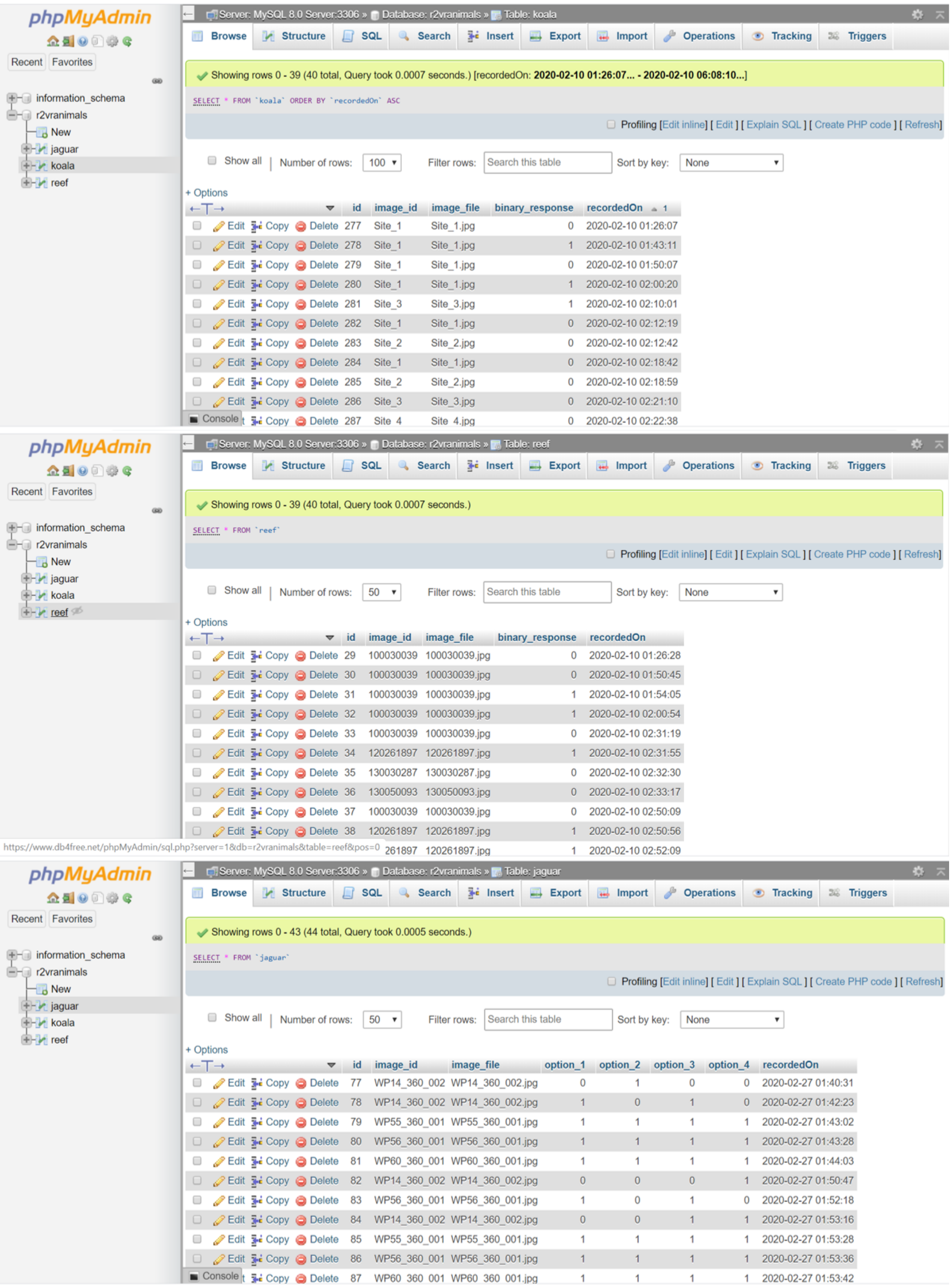

Figure S1: Screenshots of the data tables located online at `https://www.db4free.net/phpMyAdmin/`. The data tables are read using the function read() from the `r2vr` package.

Training Document for participants

You will be immersed in 3 different virtual ecosystems: a coral reef, a jungle, and forest. For each ecosystem you will be asked to answer a question. You will view four images of each ecosystem, viewing a total of 12 images and answering 12 questions. This experience should last approximately 25 minutes.

There are no risks beyond normal day-to-day living associated with your participation in this project. The only risk regarding VR is that it may induce nausea. If you experience discomfort, simply put down the headset and tell the researcher. You may cease participation from the study at any time.

You will be asked to sit in a chair before giving you the hand-held VR headset. The researcher will explain how to adjust and use the headset. You will then view your first virtual ecosystem. Look around the image. When you are ready, ask the researcher to "pop the question." A question will appear within the image. Respond to the question according to the guidance given in sections below. Inform the researcher once you have answered the question. The researcher will load a new image. Repeat this process for all 12 images.

Koala

Researchers have taken photos in areas where koalas are known to be present, but do not know how large the population is. For each photo, you will need to say if there are any koalas (one or more) present. If you see a small grey, grey-brown, or grey-white lump, especially in the crotch of larger tree branches, this is likely a koala.

Jaguar

Researchers are looking for jaguar habitat within an area of the Peruvian Amazon. For each photo, select if the habitat has:

- Water: flowing water or ponds. Do not count puddles or other small amounts of water
- Dense vegetation: any patches of vegetation that would be difficult for an average adult to walk through. Patches of vegetation should look large enough that a jaguar-sized animal could be completely concealed in them
- Jaguar tracks: Look at the ground. Select this feature if you see large cat-like footprints
- Scratch marks: present on trees with a diameter larger than 15cm

Coral reef

Researchers are interested in knowing whether or not the living reefs in these images are providing shelter for fish and other wildlife. For each photo, you will need to say if the reef is structurally complex. A complex structural habitat has different shapes and forms that create micro-habitats that creatures can live in. Think like a small fish – are there lots of places to hide? Are there multiple levels of coral and is the surface uneven? If yes, the habitat is structurally complex.

r2vr functions

start()

```
# https://github.com/ACEMS/r2vr/blob/master/R/start.R
#' Start VR server
#'
#' @param LOCAL_IP Character string of local ip address (\code{IPv4}).
If unknown go to command window and enter \code{'ipconfig'}.
#'
#' @examples
#' \donttest{
#' start("YOUR-LOCAL-IP")
#' }
#' @export
start <- function(LOCAL_IP){
  animals$serve(host = LOCAL_IP)
}
```

end()

```
# https://github.com/ACEMS/r2vr/blob/master/R/end.R
#' End VR server
#'
#' @export
#'
#' @examples
#' \donttest{
#' start("192.168.43.72")
#' end()
#' }
end <- function(){
  a_kill_all_scenes()
}
```

pop()

```
# https://github.com/ACEMS/r2vr/blob/master/R/pop.R
#' Pop messages in VR scene
#'
#' @param visible Optional logical to unpop or repop. Defaults to \code{TRUE}.
#' @param question_type Optional question type from \code{"binary"}
or \code{"multivariable"}. Defaults to \code{"binary"}.
#'
#' @examples
#' \donttest{
#' ## Display messages in VR scene
#' pop()
#'
#' ## Remove messages
```

```
#' pop(FALSE)
#' }
#'
#' @export
pop <- function(visible = TRUE, question_type = "binary"){

  if(question_type == "binary"){
    show_messages <- list(
      a_update(id = "questionPlane",
               component = "visible",
               attributes = TRUE),
      a_update(id = "yesPlane",
               component = "visible",
               attributes = TRUE),
      a_update(id = "noPlane",
               component = "visible",
               attributes = TRUE),
      a_update(id = "yesPlaneBoundary",
               component = "visible",
               attributes = TRUE),
      a_update(id = "noPlaneBoundary",
               component = "visible",
               attributes = TRUE)
    )
  }
  if(question_type == "multivariable"){
    show_messages <- list(
      a_update(id = "questionPlane",
               component = "visible",
               attributes = TRUE),
      a_update(id = "option1Plane",
               component = "visible",
               attributes = TRUE),
      a_update(id = "option3Plane",
               component = "visible",
               attributes = TRUE),
      a_update(id = "option4Plane",
               component = "visible",
               attributes = TRUE),
      a_update(id = "option2Plane",
               component = "visible",
               attributes = TRUE),
      a_update(id = "postPlane",
               component = "visible",
               attributes = TRUE),
      a_update(id = "postPlaneBoundary",
               component = "visible",
               attributes = TRUE),
      a_update(id = "option1Boundary",
               component = "visible",
```

```
              attributes = TRUE),
      a_update(id = "option3Boundary",
              component = "visible",
              attributes = TRUE),
      a_update(id = "option4Boundary",
              component = "visible",
              attributes = TRUE),
      a_update(id = "option2Boundary",
              component = "visible",
              attributes = TRUE)
    )

  }

  visible_message <- change_message(show_messages, visible)
  animals$send_messages(visible_message)
}
```

go()

```
# https://github.com/ACEMS/r2vr/blob/master/R/go.R
#' Go to next VR scene
#'
#' @param image_paths Character string of image paths from current
working directory.
#' @param index Optional numeric input to select a specific image.
#' @param question_type Optional question type from \code{"binary"}
or \code{"multivariable"}. Defaults to \code{"binary"}.
#'
#' @examples
#' \donttest{
#' image_paths <- c("img1.jpg", "img2.jpg", "img3.jpg")
#'
#' go(image_paths, 2)
#' go(image_paths, 3)
#' }
#'
#' @export
go <- function(image_paths, index = NA, question_type = "binary"){

  white <- "#ffffff"

  # Current image number
  if(is.na(index)) { CONTEXT_INDEX <- 1 }
  if(!is.na(index)){ CONTEXT_INDEX <- index }

  animal_contexts <- paste("img", seq(1,length(image_paths),1), sep="")

  # TODO: Refactor as an argument?
  context_rotations <- list(list(x = 0, y = 0, z = 0),
```

```
                            list(x = 0, y = 0, z = 0),
                            list(x = 0, y = 0, z = 0),
                            list(x = 0, y = 0, z = 0))

if(is.na(index)) {
  CONTEXT_INDEX <<- ifelse(CONTEXT_INDEX > length(animal_contexts) - 1,
                           yes = 1,
                           no = CONTEXT_INDEX + 1)
}

next_image <- animal_contexts[[CONTEXT_INDEX]]
print(next_image)


pop(FALSE, question_type )

if(question_type == "binary"){
  setup_scene <- list(
    a_update(id = "canvas3d",
             component = "material",
             attributes = list(src = paste0("#","img1"))),
    a_update(id = "canvas3d",
             component = "src",
             attributes = paste0("#","img1")),
    a_update(id = "canvas3d",
             component = "rotation",
             attributes = list(x = 0, y = 0, z = 0)),
    a_update(id = "canvas3d",
             component = "class",
             attributes = image_paths[1]),
    a_update(id = "yesPlane",
             component = "color",
             attributes = white),
    a_update(id = "noPlane",
             component = "color",
             attributes = white))
} else {
  setup_scene <-  list(
    a_update(id = "canvas3d",
             component = "material",
             attributes = list(src = paste0("#",next_image))),
    a_update(id = "canvas3d",
             component = "src",
             attributes = paste0("#",next_image)),
    a_update(id = "canvas3d",
             component = "rotation",
             attributes = context_rotations[[CONTEXT_INDEX]]),
    a_update(id = "canvas3d",
             component = "class",
             attributes = img_paths[CONTEXT_INDEX]),
```

```
      a_update(id = "option1Plane",
               component = "color",
               attributes = white),
      a_update(id = "option3Plane",
               component = "color",
               attributes = white),
      a_update(id = "option4Plane",
               component = "color",
               attributes = white),
      a_update(id = "option2Plane",
               component = "color",
               attributes = white),
      a_update(id = "postPlane",
               component = "color",
               attributes = white))
  }

  for(jj in 1:length(setup_scene)){
    if(setup_scene[[jj]]$id == "canvas3d"){
      if(setup_scene[[jj]]$component == "material"){
        setup_scene[[jj]]$attributes <- list(src = paste0("#",next_image))
      }
      if(setup_scene[[jj]]$component == "src"){
        setup_scene[[jj]]$attributes <- paste0("#",next_image)
      }
      if(setup_scene[[jj]]$component == "rotation"){
        setup_scene[[jj]]$attributes <- context_rotations[[CONTEXT_INDEX]]
      }
      if(setup_scene[[jj]]$component == "class"){
        setup_scene[[jj]]$attributes <- image_paths[CONTEXT_INDEX]
      }
    }
  }

  # TODO: Consider passing this in as an argument as binary and multiple
  # selections differ
  animals$send_messages(setup_scene)
}
```

read()

```
# https://github.com/ACEMS/r2vr/blob/master/R/read.R
#' Get data from database with API GET request
#'
#' @param url url for database.
#'
#' @return
#'
#' @examples
#' \donttest{
```

```
#' data.df <- read("https://test-api-koala.herokuapp.com/koala")
#' }
#' @export
read <- function(url){
  # Deserialize the payload so data can be read and converted
  # from JSON to data frame
  data.df <<- jsonlite::fromJSON(httr::content(httr::GET(url), "text"),
  flatten = TRUE)

  return(data.df)
  }
```
